# Leptin levels are associated with coronary artery calcification in patients with advanced prostate cancer

**DOI:** 10.1093/oncolo/oyae308

**Published:** 2024-11-18

**Authors:** Efstratios Koutroumpakis, Neha Venkatesh, Ana Aparicio, Juhee Song, Theocharis Panaretakis, Anita Deswal, Christopher J Logothetis, Daniel E Frigo, Andrew W Hahn

**Affiliations:** Department of Cardiology, Division of Internal Medicine, The University of Texas MD Anderson Cancer Center, Houston, TX 77030, United States; Department of Medicine, Baylor College of Medicine, Houston, TX 77030, United States; Department of Genitourinary Medical Oncology, Division of Cancer Medicine, The University of Texas MD Anderson Cancer Center, Houston, TX 77030, United States; Department of Biostatistics, The University of Texas MD Anderson Cancer Center, Houston, TX 770304, United States; Department of Genitourinary Medical Oncology, Division of Cancer Medicine, The University of Texas MD Anderson Cancer Center, Houston, TX 77030, United States; Department of Cardiology, Division of Internal Medicine, The University of Texas MD Anderson Cancer Center, Houston, TX 77030, United States; Department of Genitourinary Medical Oncology, Division of Cancer Medicine, The University of Texas MD Anderson Cancer Center, Houston, TX 77030, United States; Department of Genitourinary Medical Oncology, Division of Cancer Medicine, The University of Texas MD Anderson Cancer Center, Houston, TX 77030, United States; Department of Cancer Systems Imaging, Division of Diagnostic Imaging, The University of Texas MD Anderson Cancer Center, Houston, TX 77030, United States; Department of Genitourinary Medical Oncology, Division of Cancer Medicine, The University of Texas MD Anderson Cancer Center, Houston, TX 77030, United States

**Keywords:** prostate cancer, coronary heart disease, adiposity, leptin

## Abstract

**Background:**

Convergent data suggest that advanced prostate cancer and coronary heart disease (CHD) share biological vulnerabilities that may be linked to adiposity. Here we explore whether leptin, as a marker and mediator of adiposity, could link prostate cancer to CHD.

**Methods:**

Patients with metastatic castration-resistant prostate cancer (mCRPC) enrolled in a phase II trial (NCT02703623) studying androgen deprivation therapy, abiraterone, prednisone, and apalutamide were eligible if they had plasma and a chest CT scan available. Coronary artery calcium (CAC) scores and adipokine levels were measured upon enrollment.

**Results:**

Of 164 patients, 87% were white. The mean age was 65.6 ± 7.5 years, 88% were either overweight or obese, 59% had hypertension, 48% had hyperlipidemia (HLD), 20% had type 2 diabetes mellitus, and 41% were former or current smokers. Coronary calcifications were found in 115 patients (70%). Among 47 patients with non-contrast chest CT scans, the median total CAC score was 133 AU (IQR 22.6-704.6). Four patients (9%) had a score of 0 AU (low risk) and 24 (51%) scores ≥100 AU, associated with high risk for major adverse cardiovascular events. Leptin levels correlated positively with the right coronary artery (RCA) CAC score [Pearson correlation coefficient (*ρ*) = 0.3715 (*P* = .0142)]. In a multivariate logistic regression analysis, older age, HLD, and higher leptin levels were independently associated with RCA calcification and a higher number of calcified coronary arteries.

**Conclusion:**

Among men with mCRPC, there was a high burden of CHD, and higher leptin levels were associated with coronary atherosclerosis independently of traditional cardiac risk factors.

**Implications for practice**

In this prospective study of men with metastatic castration-resistant prostate cancer, the prevalence of cardiovascular risk factors and coronary atherosclerosis was high. Elevated leptin levels, which correlated with higher adiposity indices, were significantly associated with calcification in the right coronary artery and the number of calcified coronary arteries, independently of traditional cardiovascular risk factors. This analysis will inform future studies that aim to mitigate the risk of adverse cardiovascular events among patients with advanced prostate cancer treated with hormone therapy.

## Introduction

Prostate cancer, the most common cancer among men, is emblematic of malignant diseases that have now become chronic diseases. Even though survival is age-related and grade-dependent, men with potentially lethal prostate cancer still frequently survive beyond a decade from diagnosis.^1^ Consequently, the prolonged survival rates have emphasized the treatment of competing causes of morbidity and mortality, particularly cardiovascular disease, the leading cause of non-cancer-related death in this population.^2^

Adiposity and adiposity-associated lifestyle have been associated with an increased risk of advanced prostate cancer as well as atherosclerotic coronary heart disease (CHD) and may biologically explain the overlap between the 2 conditions.^3-5^ Furthermore, adiposity influences the levels of sex steroids, locally and systemically (↓testosterone ↑estrogen levels), and thus may affect response to and toxicity from hormone therapy (HT), the backbone of advanced prostate cancer treatment.^6,7^

Body mass index (BMI) has been traditionally used for the quantification of obesity, however, it is an imperfect surrogate for adiposity.^4^ Contemporary CT techniques allow for objective body composition assessment including quantification of visceral and subcutaneous fat.^8^ In addition to quantifying body composition and volume of adiposity, serum adipokines are widely studied cellular signaling proteins produced by adipose tissue.^9^ Of particular interest is the adipokine leptin, which is secreted from white adipose cells and has been shown to stimulate vascular inflammation, oxidative stress, and vascular smooth muscle hypertrophy that contribute to the pathogenesis of atherosclerosis and CHD.^10^ Additionally, emerging evidence has demonstrated the direct and indirect biological effects of leptin in regulating cancer proliferation, metastasis, angiogenesis, and chemoresistance.^11^ Leptin has been implicated in the pathogenesis and/or progression of a variety of malignancies including breast cancer, thyroid cancer, endometrial cancer, and gastrointestinal malignancies.^12-15^ In a recent meta-analysis of 31 studies, leptin was also associated with aggressiveness of prostate cancer.^16^

We propose that the accurate assessment of the amount of baseline adiposity and its biological activity, as measured by adipokines, in patients with advanced prostate cancer will shed light on the biological connection between advanced prostate cancer and CHD. Understanding this biological link will allow for precise patient risk stratification and personalization of cancer treatment accounting for competing comorbidities and potential risk for toxicity. The specific aim of this study was to screen for associations between leptin and specific cardiac outcomes in patients with metastatic prostate cancer. The overall goal of our effort is to identify associations supporting the hypothesis that adiposity is a functional link connecting advanced prostate cancer with CHD.

## Methods

### Patient population

We conducted a post hoc analysis of a modular, open-label, randomized, single-institution, phase II trial (DynAMo trial; NCT02703623) studying 8 weeks of androgen deprivation therapy (ADT), abiraterone acetate, prednisone, and apalutamide (AAPA) with or without ipilimumab or cabazitaxel and carboplatin for the treatment of men with metastatic castration-resistant prostate cancer (mCRPC).^17^ Men enrolled in this trial were eligible to be included in our analysis if they had available, pretreatment plasma as well as a chest and an abdomen/pelvis CT scan. CT scans chronologically closest to the enrollment date were used. Demographic and clinical data were prospectively collected as part of the clinical trial. Baseline cardiovascular risk factors, medications, and laboratory values were retrospectively collected. This study was approved by the MD Anderson Cancer Center (MDACC) Institutional Review Board.

### Body composition and serum leptin levels

Body composition was measured using the mid-L3 slice of a computed tomography (CT) scan of the abdomen and pelvis that was obtained prior to initiating AAPA and after exposure to ADT using the SliceOmatic image analysis software (Tomovision, Quebec, Canada). Body composition measures included visceral, subcutaneous, and total adipose tissue volume (VAT, SAT, and TAT, respectively) as well as skeletal muscle mass (SMM), skeletal muscle density (SMD), and intramuscular adipose tissue (IMA) volume. Body composition measures were normalized for height in meters squared (m^2^). To align with the timing of body composition measurement, non-fasting plasma samples obtained prior to AAPA were utilized for the measurement of leptin levels, which were performed using the Quantibody Human Obesity Array Q3 (Raybiotech).

### Coronary artery calcification

Coronary calcifications were qualitatively and quantitatively assessed. Standard-of-care, non-ECG gated, chest CT scans with or without contrast were reviewed, and the presence or absence of coronary calcification in each coronary artery was marked. Chest CT scans with contrast did not allow the measurement of coronary artery calcium (CAC) scores. For patients with available chest CT scans without contrast, CAC scoring was performed following the 2016 SCCT/STR guidelines.^18^ Non-contrast CT scans were uploaded into a workstation (Syngo.via, Siemens Healthcare). The CAC score was quantified in each study using the CT Ca Scoring application from the CT Cardiac package of Syngo.Via (Siemens Healthcare). Each chest CT study was processed by a cardiologist, board-certified in cardiac CT interpretation. A meticulous assessment of all coronary territories was performed for CAC assessment, making sure that mitral annular calcification, valvular calcification, and cardiac implants did not interfere with the CAC calculation. Patients with prior coronary revascularization with stents were excluded from this analysis. Total and individual coronary artery CAC scores were collected.

### Statistical analysis

Patient demographic and baseline characteristics are summarized using descriptive statistics; mean (SD) or median (IQR) for continuous variables and frequency (%) for categorical variables. Pearson correlation analysis was conducted among body composition, leptin, and coronary calcium scores. Univariate and multivariate logistic regression models were fitted to assess the significance of each covariate on coronary calcification. Linear regression models were fitted to assess the significance of each covariate on the number of calcified arteries. Based on univariate models (*P* < .1), we selected candidates to consider in multivariate models and then significant covariates were selected by stepwise selection method. A *P*-value less than .05 was used to assess statistical significance. SAS 9.4 (SAS Institute Inc.) was used for data analysis.

## Results

**Figure 1.**
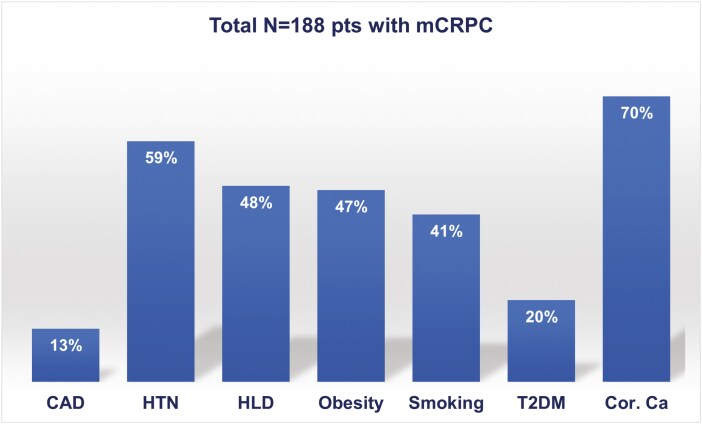
Prevalence of cardiovascular risk factors, coronary calcifications, and clinical coronary artery disease among 188 patients enrolled in the DynAMo trial (percentages of cardiovascular risk factors and coronary calcifications refer to a total number of 164 patients, after excluding those with coronary revascularization for CAD). Abbreviations: CAD, coronary artery disease; Cor. Ca, coronary calcifications; HLD, hyperlipidemia; HTN, hypertension; T2DM, type 2 diabetes mellitus

A total of 188 patients with mCRPC were initially screened. Of 188 patients, 24 (13%) had undergone coronary revascularization for CHD and were excluded from the current analysis. Out of the remaining 164 patients, 87% were white, 8% black or African American, and 10% Hispanic. Mean ± SD age and mean ± SD BMI were 65.6 ± 7.5 years and 30.2 ± 4.7 kg/m^2^, respectively, with 88% being overweight or obese [(BMI 25-29.9 kg/m^2^ 41%), BMI ≥ 30 kg/m^2^ 47%, respectively)]. Cardiovascular risk factors were common, with 59% of patients having a history of hypertension (HTN), 48% hyperlipidemia (HLD), 41% being former (34%) or current (7%) smokers, and 20% having a history of type 2 diabetes mellitus (T2DM; Table 1, Figure 1).

**Table 1.** Demographical and baseline clinical characteristics of patients with metastatic castration-resistant prostate cancer.

	*N* = 164
Age in years, mean ± SD	65.55 ± 7.49
Race, *N* (%)	
- White	137(86.7%)
- Black/African American	12(7.6%)
- Other	9(5.7%)
- Unknown	6
Ethnicity, *N* (%)	
- Hispanic or Latino	16(9.9%)
- Non-Hispanic	145(90.1%)
- Unknown	3
BMI in kg/m^2^, mean ± SD	30.18 ± 4.71
BMI_group, *N* (%)	
- <25 kg/m^2^	20(12.2%)
- 25-29.9 kg/m^2^	67(40.9%)
- ≥30 kg/m^2^	77(47%)
HTN, *N* (%)	96(58.5%)
HLD, *N* (%)	79(48.2%)
T2DM, *N* (%)	32(19.5%)
Smoker, *N* (%)	
- Current	11(6.7%)
- Former	56(34.1%)
- Never	97(59.1%)
HgA1C, median (IQR)^*^	5.7(5.4-6.1)
LDL, mean ± SD	109.15 ± 36.15
Cholesterol, mean ± SD	191.45 ± 41.33
HDL, mean ± SD	53.97 ± 16.03
Triglyceride, median (IQR)	133.5(99.5-168)
Troponin, median (IQR)	18.5(10-29.5)
NTproBNP, median (IQR)	365(123-711)
Creatinine, median (IQR)	0.95(0.83-1.06)
Albumin, median (IQR)	4.2(4-4.3)
CRP, median (IQR)	6.73(2.63-50.44)
PSA, median (IQR)	4.1(1.2-14.45)
Statin, *N* (%)	69(42.1%)
Aspirin, *N* (%)	52(31.7%)
ARB, *N* (%)	34(20.7%)
ACEi, *N* (%)	54(32.9%)
Metformin, *N* (%)	36(22%)

^*^Labs performed chronologically closest to the date of enrollment to DynAMo trial were included.

ACEi, angiotensin converting enzyme inhibitor, ARB, angiotensin receptor blocker, BMI, body mass index, HDL, high density lipoprotein, HgA1C, hemoglobin A1C, HLD, hyperlipidemia, HTN, hypertension, IQR, interquartile range, LDL, low density lipoprotein, SD, standard deviation, T2DM, type 2 diabetes mellitus

A total of 152 patients had measurements of serum leptin at the time of enrollment. The median leptin levels were 6270.96 pg/mL (IQR 3940.95-7042.09). The median time from the enrollment of the patient to the CT scan of the abdomen and pelvis was 55 days (IQR 55-56). The body composition measurements of the patients included in this analysis are presented in Table 2. As shown in our previous work, there was a significant positive correlation among VAT, SAT, TAT, and IMA with leptin levels, whereas there was a significant negative correlation between SMD and leptin levels (Supplementary Table S1).^19^

**Table 2.** Leptin levels and computed tomography-based measures of body composition and coronary heart disease among patients with metastatic castration-resistant prostate cancer.

	N	
Leptin (pg/mL), median (IQR)	152	6270.96 (3940.95-,7042.09)
Log(leptin), mean ± SD	152	3.69 ± 0.26
*Measures of adiposity*		
Time from enrollment to CT abdomen and pelvis in days, median (IQR)	164	55(55-56)
TAT_index, mean ± SD	163	150.79 ± 58.82
VAT_index, median (IQR)	163	68.1(43.4-105.91)
SAT_index, median (IQR)	163	73.25(54.86-93.55)
SMM_index, mean ± SD	163	49.89 ± 6.96
SMD_hu, mean ± SD	160	38.37 ± 7.98
IMA_index, median (IQR)	163	4.75(3.01-6.56)
*Measures of coronary calcifications*		
Time from enrollment to CT chest in days, median (IQR)	164	56(55-58)
Evidence of coronary calcifications, N (%)	164	115(70.1%)
Number of coronary arteries affected, median (IQR)	164	2(0-3)
LM_calcification, *N* (%)	164	50(30.5%)
LAD_calcification, *N* (%)	164	106(64.6%)
LM/LAD calcification, *N* (%)	164	109 (66.5%)
LCx_calcification, *N* (%)	164	66(40.2%)
RCA_calcification, *N* (%)	164	58(35.4%)

Abbreviations: IMA, intramuscular adipose tissue; IQR, interquartile range; LAD, left anterior descending coronary artery; LCx, left circumflex coronary artery; LM, left main coronary artery; RCA, right coronary artery; SAT, subcutaneous adipose tissue volume; SD, standard deviation; SMD, skeletal muscle density; SMM, skeletal muscle mass; TAT, total adipose tissue volume; VAT, visceral adipose tissue volume.

The median time from the enrollment of the patient to the CT scan of the chest was 56 days (IQR 55-58). Coronary calcifications were identified in 115 patients (70%) with a median number of coronary arteries affected of 2 (IQR 0-3). Coronary calcifications in the left main (LM) coronary artery were present in 31%, left anterior descending (LAD) artery in 65%, left circumflex (LCx) artery in 40%, and right coronary artery (RCA) in 35% of the patients (**Table 2**). Twenty-eight patients (17%) had diffuse coronary calcifications in all coronary arteries. A total of 47 patients had undergone a CT scan of the chest without contrast which allowed the calculation of coronary artery calcium (CAC) scores. The median total CAC score was 133 AU (IQR 22.6-704.6). Out of 47, 4 patients (9%) had a total score of 0 AU, 19 (40%) had a total score of 1-99 AU, consistent with mild amount of atherosclerotic plaque and low risk for major adverse cardiovascular events (MACE), 10 (21%) had a total score of 100-399 AU, consistent with moderate amount of atherosclerotic plaque and moderate risk for MACE and 14 (30%) had a total score of 400 or more AU, consistent with large amount atherosclerotic plaque and high risk for MACE.

Among 43 patients with available leptin levels and CAC scores, there was a positive correlation between leptin levels and RCA CAC score [*ρ* = 0.3715 (*P* = .0142)] while there was a trend toward a positive correlation between leptin levels and total CAC score [*ρ* = 0.2727 (*P* = .0769)]. No statistically significant correlations were noted between leptin levels and calcifications in the LM, LAD, or LCx arteries (Table 3**).**

**Table 3.** Correlation of leptin levels with coronary calcium scores among 43 patients with metastatic castration-resistant prostate cancer.

	Pearson correlation coefficients (*P*-value) *N* = 43
	Leptin (ng/mL)
Total calcium score	0.27268 (*P* = .0769)
LM calcium score	0.05607 (*P* = .7210)
LAD calcium score	0.20930 (*P* = .1780)
LCx calcium score	0.02096 (*P* = .8939)
RCA calcium score	0.37145 (*P* = .0142)

Abbreviations: LM, left main coronary artery; LAD, left anterior descending coronary artery; LCx, left circumflex coronary artery; RCA, right coronary artery.

A logistic regression analysis was performed to identify demographic and clinical characteristics that are associated with calcifications in the RCA. In the multivariate analysis, older age, history of HLD, and higher leptin levels were significantly and independently associated with RCA calcification (Table 4). Of note, measures of adiposity were significantly associated with RCA calcification in the univariate analysis, but not in the multivariate analysis. In a separate multivariate linear regression analysis, older age, history of HLD, and higher leptin levels were also significantly and independently associated with a higher number of calcified coronary arteries (Supplementary Table S2).

**Table 4.** Association of baseline characteristics with RCA calcification (yes (*n* = 58) vs no (*n* = 106)).

		Univariate logistic regression		Multivariate logistic regression^1^	
Covariate	Level	OR (95% CI)	*P*-value	OR (95% CI)	*P*-value
Age at registration (yrs)	In 1 Unit Change	1.07(1.02-1.13)	**.0036**	1.07(1.01-1.12)	.0149
BMI (kg/m^2^)	In 1 Unit Change	1.09(1.02-1.17)	**.0150**		
BMI group	<25 kg/m^2^	1.00			
	25-29.9 kg/m^2^	3.16(0.84-11.90)	**.0886**		
	>=30 kg/m^2^	3.82(1.03-14.14)	**.0449**		
Race	White	1.00			
	Black	0.35(0.07-1.65)	.1841		
	Other	0.87(0.21-3.63)	.8485		
Ethnicity	Hispanic Or Latino	1.00			
	Non-Hispanic	1.73(0.53-5.63)	.3642		
HTN	Yes	2.24(1.13-4.42)	**.0207**		
HLD	Yes	2.4(1.24-4.63)	**.0091**	2.73(1.29-5.79)	.0087
Smoker	Never	1.00			
	Current	1.77(0.50-6.26)	.3729		
	Former	1.38(0.69-2.73)	.3596		
T2DM	Yes	2.14(0.98-4.69)	**.0567**		
Statin	Yes	2.87(1.48-5.57)	**.0017**		
ASA	Yes	0.84(0.42-1.69)	.6258		
ARB	Yes	2.96(1.36-6.41)	**.0061**		
ACEi	Yes	1.41(0.72-2.77)	.3140		
Metformin	Yes	1.9(0.90-4.03)	**.0949**		
A1C	In 1 Unit Change	0.91(0.60-1.38)	.6479		
LDL	In 1 Unit Change	1(0.99-1.02)	.6523		
Cholesterol	In 1 Unit Change	1(0.99-1.02)	.5929		
HDL	In 1 Unit Change	1.01(0.97-1.04)	.7450		
Triglyceride	In 1 Unit Change	1(0.99-1.01)	.9732		
Troponin	In 1 Unit Change	1.01(0.97-1.05)	.6584		
NTproBNP	In 1 Unit Change	1(1.00-1.00)	.7567		
Creatinine	In 1 Unit Change	2.18(0.52-9.15)	.2871		
Albumin	In 1 Unit Change	0.44(0.13-1.50)	.1896		
CRP	In 1 Unit Change	1.01(1.00-1.01)	**.0690**		
PSA	In 1 Unit Change	0.99(0.98-1.00)	**.0825**		
Leptin (ng/mL)	In 1 Unit Change	1.42(1.17-1.71)	**.0004**	1.45(1.20-1.76)	.0002
Leptin (pg/mL)	In 10 Unit Change	1.003(1.002-1.005)	**.0004**	1.004(1.002-1.006)	.0002
log(leptin) (pg/ml))	In 1 Unit Change	35.45(4.20-299.22)	.0010		
SMM index	In 1 Unit Change	1(0.96-1.05)	.9347		
SMD (HU)	In 1 Unit Change	0.94(0.90-0.99)	**.0079**		
IMA index	In 1 Unit Change	1.17(1.04-1.32)	**.0108**		
VAT index	In 1 Unit Change	1.02(1.01-1.03)	**.0002**		
SAT index	In 1 Unit Change	1.01(1.00-1.02)	**.0806**		
TAT index	In 1 Unit Change	1.01(1.00-1.02)	**.0008**		

^1^Multivariate logistic model is selected by stepwise selection, initially including age, leptin, SMU_HU, TAT index, BMI, HTN, HLD, and T2DM (TAT index is highly correlated with VAT index and SAT index. Therefore, only TAT index is included in the model). Bold indicates a p-value of less than 0.05.

**Figure 2.**
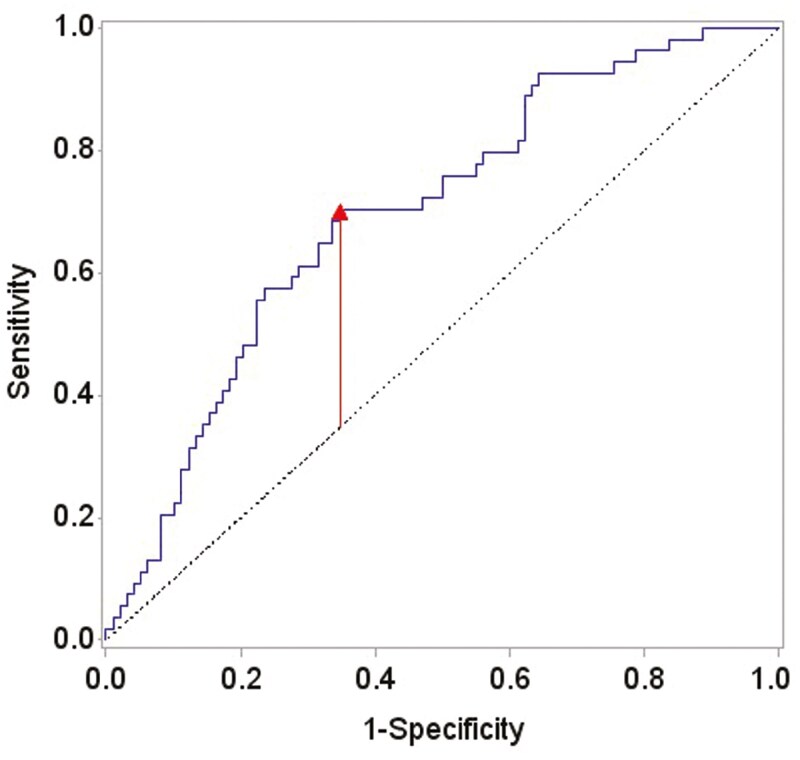
Based on the maximum Youdan index criterion, leptin cutoff value of 6.35 ng/mL was selected. For this cutoff value, sensitivity is 0.70, specificity is 0.65, PPV is 0.53, and NPV is 0.8 (for leptin, median is 6.27 and mean is 5.55). AUC of ROC curve: 0.70 (95% CI, 0.61-0.78).

Based on the maximum Youdan index, a leptin cutoff value of 635 pg/mL was selected as the optimal level for predicting RCA calcification (Figure 2). Leptin levels above 635 pg/mL were associated with RCA calcification with a sensitivity of 70%, specificity of 65%, PPV of 53%, and NPV of 80% [AUC of ROC curve: 0.70 (95% CI, 0.61-0.78)].

During a median follow-up of 5.9 years, 10 patients without prior CHD (6%) developed a major adverse cardiovascular outcome (6 patients developed a non-fatal stroke, 2 patients had non-fatal myocardial infarction, and 2 patients had unstable angina). The small number of MACE in this study did not allow for meaningful statistical correlations with leptin levels and coronary calcium scores.

## Discussion

To the best of our knowledge, this is the first study examining the correlation of CT measures of adiposity and leptin levels with calcified atherosclerotic CHD in patients with mCRPC. In this prospective study of men with mCRPC, we found that the prevalence of cardiovascular risk factors and coronary atherosclerosis was high. In addition, we showed that elevated leptin levels, which correlated with higher adiposity indices, were significantly associated with calcification in the right coronary artery and a number of calcified coronary arteries, independently of traditional cardiovascular risk factors.

Several previous studies report a high prevalence of cardiovascular risk factors and cardiovascular disease among patients with prostate cancer.^20-22^ Based on the RADICAL-PC study, a prospective study of almost 2500 men with prostate cancer, more than half of the patients had HLD and were active or former smokers, over 1/3 had HTN and were obese, and almost 20% of them had T2DM. Furthermore, 22% of patients had established cardiovascular disease at baseline while 1/3 of them were at high risk for developing myocardial infarction, stroke, or cardiovascular death based on the Framingham risk assessment tool.^20^ Our findings are in line with the above study and further confirm the high burden of cardiovascular comorbidities among patients with prostate cancer. In addition to the above study which used traditional cardiovascular risk factors and the Framingham risk assessment tool, we quantified the burden of CHD using the presence of coronary calcifications on CT imaging, which has emerged as the single best predictor of adverse coronary artery events.^23^ We showed that more than two-thirds of patients in our cohort had evidence of coronary calcifications, while more than half had a CAC score above 100 AU and one-third had a CAC score above 400 AU, consistent with very high risk. Estimation of CAC using standard-of-care CT scans is a quick, cost-effective way to stratify patients with prostate cancer based on their cardiovascular risk, and our findings suggest it may be considered as part of the routine care of men on HT.^24^

The incidence of prostate cancer and CHD increases with age, yet convergent data suggest they may have shared biologic vulnerabilities and are more than mere age-related associations.^25^ Low androgen levels and androgen signaling have been associated with adiposity and adiposity-linked cardiometabolic illnesses including HTN, HLD, and T2DM.^26-29^ Additionally, low initial concentrations of testosterone have been associated with poor prognosis in men with advanced prostate cancer, without a clear mechanistic link.^30^ Further, ADT, the mainstay of advanced prostate cancer treatment, leads to increases in adiposity and adiposity-related metabolic derangements. On the contrary, statins, the main preventive cardiovascular therapy that alters adiposity-related metabolic derangements, have been shown to improve response to HT in early studies, delay cancer progression, and reduce the risk of prostate-cancer-specific mortality.^31-34^ The data on other cardiovascular therapies that target adiposity-related metabolic derangements, such as metformin are conflicting. Even though some early studies had shown a possible benefit of metformin toward prostate cancer-specific outcomes,^35,36^ the recent Metformin Active Surveillance Trial (MAST trial) failed to show delayed cancer progression among treated patients with low-risk prostate cancer compared to controls.^37^ Nonetheless, the above observations taken all together, suggest that adiposity may be a key biological link among androgen signaling, prostate cancer progression, and acceleration of cardiovascular disease development. The relationship among CHD, adiposity, and androgen signaling is beyond the scope of this study, but our group is studying this on multiple fronts.

Our group and others have objectively quantified body composition and body composition changes in patients with different stages of prostate cancer and have reported associations with response to androgen signaling inhibition and toxicity as well as overall survival.^19,38,39^ In addition to quantifying body composition and volume of adiposity, serum adipokines are widely studied cellular signaling proteins produced by adipose tissue.^40^ Leptin, an adipokine secreted specifically from white adipose cells, known to primarily regulate appetite, has been shown to stimulate vascular inflammation, oxidative stress, and vascular smooth muscle hypertrophy, all important pathogenetic steps of atherosclerosis and CHD.^41^ Furthermore, accumulating evidence reports a key pathophysiological role of leptin in prostate cancer progression and its association with advanced disease.^42^ Our current study, showed a significant positive correlation of leptin levels with adiposity and the burden of atherosclerotic CHD in patients with mCRPC, which was independent of traditional cardiovascular risk factors. Of note, a direct correlation between leptin levels and MACE was not feasible due to the small number of MACE in this study. However, leptin levels did correlate with coronary calcium score, which has been shown in large studies to be the single strongest predictor of MACE compared to traditional cardiovascular risk factors.^43^ The findings of this study support the hypothesis that adiposity and leptin are key biological links between prostate cancer and CHD. This is particularly important in the era of new, effective medical therapies that reduce adiposity and implies that adiposity might represent a novel therapeutic target for patients with prostate cancer.^44,45^ Finally, based on the results of our study, serum leptin levels could also be used as a marker for patient risk stratification and personalization of prostate cancer treatment by guiding preventive cardiovascular therapies such as statins.

This study has several limitations. First, the sample size of the study is small, which in the setting of low statistical power may explain the lack of significant correlation between leptin levels and CAC scores of the coronary arteries other than the RCA, as well as the total CAC score. However, it is also possible that the significant correlation we detected between leptin levels and RCA CAC score represents an alpha error and no true correlation exists. Furthermore, a large portion of patients suffered from cardiovascular comorbidities at baseline, including HTN, HLD, and obesity, which have been linked to leptin levels and CAC scores and might have affected the results of our univariate and multivariate analysis. Additionally, the number of MACE in this study was small. This is likely related to the incomplete and relatively short follow up in the setting of a large portion of patients being treated outside our tertiary cancer center for their non-cancer-related disease. The small event rate in this study did not allow for direct correlations between leptin levels and MACE. Furthermore, the CT scans used in this study were scans done at the discretion of the treating clinicians and as part of the standard of care of the patients. Therefore, this may have introduced selection bias as patients who were sicker were more likely to have undergone CT scans of the chest, abdomen, and pelvis. Finally, CAC scores were calculated using non-ECG-gated CT scans and likely underrepresent the true CAC scores of the patients. Despite its limitations, this is the first prospective study examining a potential functional link connecting advanced prostate cancer and CHD, through adiposity and leptin.

In conclusion, in this post-hoc analysis of men with mCRPC, the burden of atherosclerotic CHD was high and correlated significantly with elevated leptin levels. Our findings support the hypothesis that a subset of patients with advanced prostate cancer and CHD share a biologic vulnerability linked by adiposity and leptin. This analysis will inform future studies that aim to mitigate the risk of adverse cardiovascular events among patients with advanced prostate cancer treated with HT.

## Supplementary material

Supplementary material is available at *The Oncologist* online.

oyae308_suppl_Supplementary_Tables_S1-2

## Data Availability

Data are available upon request submitted to the corresponding author.

## References

[1] Song P , Wang J, Shu M, et al Prognosis of men with high-risk prostate cancer stratified by risk factors: a population-based retrospective cohort study. Transl Cancer Res. 2020;9:6013-6025. https://doi.org/10.21037/tcr-20-1578 35117213 PMC8797365

[2] Elmehrath AO , Afifi AM, Al-Husseini MJ, et al Causes of death among patients with metastatic prostate cancer in the US from 2000 to 2016. JAMA Network Open. 2021;4:e2119568-e2119568. https://doi.org/10.1001/jamanetworkopen.2021.19568 34351403 PMC8343467

[3] Discacciati A , Orsini N, Wolk A. Body mass index and incidence of localized and advanced prostate cancer—a dose-response meta-analysis of prospective studies. Ann Oncol. 2012;23:1665-1671. https://doi.org/10.1093/annonc/mdr603 22228452

[4] Powell-Wiley TM , Poirier P, Burke LE, et al; American Heart Association Council on Lifestyle and Cardiometabolic Health; Council on Cardiovascular and Stroke Nursing; Council on Clinical Cardiology; Council on Epidemiology and Prevention; and Stroke Council. Obesity and cardiovascular disease: a scientific statement from the American Heart Association. Circulation. 2021;143:e984-e1010. https://doi.org/10.1161/CIR.0000000000000973 33882682 PMC8493650

[5] Pulliam TL , Awad D, Han JJ, et al Systemic ablation of Camkk2 impairs metastatic colonization and improves insulin sensitivity in TRAMP mice: evidence for cancer cell-extrinsic CAMKK2 functions in prostate cancer. Cells. 2022;11:1890. https://doi.org/10.3390/cells11121890 35741020 PMC9221545

[6] Rocha-Rodrigues S , Matos A, Afonso J, et al Skeletal muscle-adipose tissue-tumor axis: molecular mechanisms linking exercise training in prostate cancer. Int J Mol Sci. 2021;22:4469. https://doi.org/10.3390/ijms22094469 33922898 PMC8123194

[7] Hahn AW , Siddiqui BA, Leo J, et al Cancer cell-extrinsic roles for the androgen receptor in prostate cancer. Endocrinology. 2023;164:bqad078. https://doi.org/10.1210/endocr/bqad078 37192413 PMC10413433

[8] Khan AI , Psutka SP, Patil DH, et al Sarcopenia and systemic inflammation are associated with decreased survival after cytoreductive nephrectomy for metastatic renal cell carcinoma. Cancer. 2022;128:2073-2084. https://doi.org/10.1002/cncr.34174 35285950

[9] Fain JN , Madan AK, Hiler ML, Cheema P, Bahouth SW. Comparison of the release of adipokines by adipose tissue, adipose tissue matrix, and adipocytes from visceral and subcutaneous abdominal adipose tissues of obese humans. Endocrinology. 2004;145:2273-2282. https://doi.org/10.1210/en.2003-1336 14726444

[10] Koh KK , Park SM, Quon MJ. Leptin and cardiovascular disease: response to therapeutic interventions. Circulation. 2008;117:3238-3249. https://doi.org/10.1161/CIRCULATIONAHA.107.741645 18574061 PMC2746068

[11] Lin TC , Hsiao M. Leptin and cancer: updated functional roles in carcinogenesis, therapeutic niches, and developments. Int J Mol Sci. 2021;22:2870. 33799880 10.3390/ijms22062870 PMC8002181

[12] Gu L , Wang C-D, Cao C, et al Association of serum leptin with breast cancer: a meta-analysis. Medicine (Baltimore). 2019;98:e14094. https://doi.org/10.1097/MD.0000000000014094 30702563 PMC6380739

[13] Zhang L , Yuan Q, Li M, et al The association of leptin and adiponectin with hepatocellular carcinoma risk and prognosis: a combination of traditional, survival, and dose-response meta-analysis. BMC Cancer. 2020;20:1167. https://doi.org/10.1186/s12885-020-07651-1 33256658 PMC7708253

[14] Refahi R , Heidari Z, Mashhadi M. Association of high serum leptin level with papillary thyroid carcinoma: a case-control study. Int J Hematol Oncol Stem Cell Res. 2023;17:210-219. https://doi.org/10.18502/ijhoscr.v17i3.13311 37817973 PMC10560642

[15] Sharma D , Saxena NK, Vertino PM, Anania FA. Leptin promotes the proliferative response and invasiveness in human endometrial cancer cells by activating multiple signal-transduction pathways. Endocr Relat Cancer. 2006;13:629-640. https://doi.org/10.1677/erc.1.01169 16728588 PMC2925427

[16] Burton AJ , Gilbert R, Tilling K, et al Circulating adiponectin and leptin and risk of overall and aggressive prostate cancer: a systematic review and meta-analysis. Sci Rep. 2021;11:320. https://doi.org/10.1038/s41598-020-79345-4 33431998 PMC7801499

[17] Aparicio AM , Tidwell RSS, Yadav SS, et al A modular trial of androgen signaling inhibitor combinations testing a risk-adapted strategy in patients with metastatic castration-resistant prostate cancer. Clin Cancer Res. 2024;30:2751-2763. 38683200 10.1158/1078-0432.CCR-23-3740 PMC11216872

[18] Hecht HS , Cronin P, Blaha MJ, et al 2016 SCCT/STR guidelines for coronary artery calcium scoring of noncontrast noncardiac chest CT scans: a report of the Society of Cardiovascular Computed Tomography and Society of Thoracic Radiology. J Cardiovasc Comput Tomogr. 2017;11:74-84. https://doi.org/10.1016/j.jcct.2016.11.003 27916431

[19] Hahn AW , Tidwell RS, Pilie PG, et al Body composition as a determinant of the therapeutic index with androgen signaling inhibition. Prostate Cancer Prostatic Dis. 2024;Online ahead of print. 10.1038/s41391-024-00870-8 39019979

[20] Leong DP , Fradet V, Shayegan B, et al Cardiovascular risk in men with prostate cancer: insights from the RADICAL PC Study. J Urol. 2020;203:1109-1116. https://doi.org/10.1097/JU.0000000000000714 31899651

[21] Sun L , Parikh RB, Hubbard RA, et al Assessment and management of cardiovascular risk factors among US veterans with prostate cancer. JAMA Network Open. 2021;4:e210070-e210070. https://doi.org/10.1001/jamanetworkopen.2021.0070 33625512 PMC7905496

[22] Moningi S , Nguyen PL. Uncontrolled cardiovascular risk factors in prostate cancer patients: are we leaving too much on the table? JACC CardioOncol. 2023;5:82-84. https://doi.org/10.1016/j.jaccao.2023.01.001 36875903 PMC9982199

[23] Nasir K , Cainzos-Achirica M. Role of coronary artery calcium score in the primary prevention of cardiovascular disease. BMJ. 2021;373:n776. https://doi.org/10.1136/bmj.n776 33947652

[24] Lopez-Mattei J , Yang EH, Baldassarre LA, et al Cardiac computed tomographic imaging in cardio-oncology: an expert consensus document of the Society of Cardiovascular Computed Tomography (SCCT). Endorsed by the International Cardio-Oncology Society (ICOS). J Cardiovasc Comput Tomogr. 2023;17:66-83. https://doi.org/10.1016/j.jcct.2022.09.002 36216699

[25] Logothetis CJ , Hahn AW. Challenging the prevailing therapeutic dogma for prostate cancer: the case for an overlap syndrome. Eur Urol. 2023;85:3-7. https://doi.org/10.1016/j.eururo.2023.04.015 37210287

[26] Harman SM , Metter EJ, Tobin JD, Pearson J, Blackman MR; Baltimore Longitudinal Study of Aging. Longitudinal effects of aging on serum total and free testosterone levels in healthy men. J Clin Endocrinol Metab. 2001;86:724-731. https://doi.org/10.1210/jcem.86.2.7219 11158037

[27] Rao PM , Kelly DM, Jones TH. Testosterone and insulin resistance in the metabolic syndrome and T2DM in men. Nat Rev Endocrinol. 2013;9:479-493. https://doi.org/10.1038/nrendo.2013.122 23797822

[28] Blaya R , Thomaz LDGR, Guilhermano F, et al Total testosterone levels are correlated to metabolic syndrome components. Aging Male. 2016;19:85-89. https://doi.org/10.3109/13685538.2016.1154523 26961662

[29] Pinthus JH , Duivenvoorden WC, Klotz L, et al Low serum testosterone in men with newly diagnosed androgen-deprivation therapy-naïve prostate cancer and its relationship to cardiovascular risk factors: a RADICAL-PC substudy. J Urol. 2022;207:1020-1028. 34978211 10.1097/JU.0000000000002384

[30] Tu H , Gu J, Meng QH, et al Low serum testosterone is associated with tumor aggressiveness and poor prognosis in prostate cancer. Oncol Lett. 2017;13:1949-1957. https://doi.org/10.3892/ol.2017.5616 28454349 PMC5403694

[31] Posielski N , Liou J-i, Khemees TA, et al The impact of statins in combination with androgen deprivation therapyin patients with advanced prostate cancer: a large observational study. Paper presented at Urologic Oncology: Seminars and Original Investigations 2019. 10.1016/j.urolonc.2018.11.017 PMC6919968 30528885

[32] Peltomaa A , Raittinen P, Talala K, et al Prostate cancer prognosis after initiation of androgen deprivation therapy among statin users. A population-based cohort study. Prostate Cancer Prostatic Dis. 2021;24:917-924. 33790420 10.1038/s41391-021-00351-2 PMC8384625

[33] Harshman LC , Wang X, Nakabayashi M, et al Statin use at the time of initiation of androgen deprivation therapy and time to progression in patients with hormone-sensitive prostate cancer. JAMA Oncol. 2015;1:495-504. https://doi.org/10.1001/jamaoncol.2015.0829 26181260 PMC5554437

[34] Pan T , Lin S-C, Lee Y-C, et al Statins reduce castration-induced bone marrow adiposity and prostate cancer progression in bone. Oncogene. 2021;40:4592-4603. https://doi.org/10.1038/s41388-021-01874-7 34127814 PMC8384136

[35] Rothermundt C , Hayoz S, Templeton AJ, et al Metformin in chemotherapy-naive castration-resistant prostate cancer: a multicenter phase 2 trial (SAKK 08/09). Eur Urol. 2014;66:468-474. https://doi.org/10.1016/j.eururo.2013.12.057 24412228

[36] Colquhoun A , Venier N, Vandersluis A, et al Metformin enhances the antiproliferative and apoptotic effect of bicalutamide in prostate cancer. Prostate Cancer Prostatic Dis. 2012;15:346-352. 22614062 10.1038/pcan.2012.16

[37] Fleshner NE , Bernardino RM, Lajkosz K, et al A randomized, double-blind, placebo-controlled trial of metformin in reducing progression among men on expectant management for low-risk prostate cancer: The MAST (Metformin Active Surveillance Trial) study. J Clin Oncol. 2024;42:LBA5002-LBA5002. https://doi.org/10.1200/jco.2024.42.17_suppl.lba5002

[38] Coletta AM , Sayegh N, Agarwal N. Body composition and metastatic prostate cancer survivorship. Cancer Treat Res Commun. 2021;27:100322. https://doi.org/10.1016/j.ctarc.2021.100322 33517236

[39] Venkatesh N , Tidwell R, Yu Y, et al Association of inherited steroidogenic genotype with body composition changes after androgen signaling inhibition (ASI) in men with biochemical recurrent (BCR), hormone-sensitive prostate cancer (HSPC). J Clin Oncol. 2024;42:108-108. https://doi.org/10.1200/jco.2024.42.4_suppl.108

[40] Kirichenko TV , Markina YV, Bogatyreva AI, et al The role of adipokines in inflammatory mechanisms of obesity. Int J Mol Sci. 2022;23:14982. https://doi.org/10.3390/ijms232314982 36499312 PMC9740598

[41] Koh KK , Park SM, Quon MJ. Leptin and cardiovascular disease. Circulation. 2008;117:3238-3249. https://doi.org/10.1161/CIRCULATIONAHA.107.741645 18574061 PMC2746068

[42] Ribeiro R , Lopes C, Medeiros R. The link between obesity and prostate cancer: the leptin pathway and therapeutic perspectives. Prostate Cancer Prostatic Dis. 2006;9:19-24. https://doi.org/10.1038/sj.pcan.4500844 16344847

[43] Hoffmann U , Massaro JM, D’Agostino RB Sr., et al Cardiovascular event prediction and risk reclassification by coronary, aortic, and valvular calcification in the Framingham Heart Study. J Am Heart Assoc. 2016;5:115.003144. 10.1161/JAHA.115.003144 PMC4802453 26903006

[44] Wilding JPH , Batterham RL, Calanna S, et al; STEP 1 Study Group. Once-weekly semaglutide in adults with overweight or obesity. N Engl J Med. 2021;384:989-1002. https://doi.org/10.1056/NEJMoa2032183 33567185

[45] Jastreboff AM , Aronne LJ, Ahmad NN, et al; SURMOUNT-1 Investigators. Tirzepatide once weekly for the treatment of obesity. N Engl J Med. 2022;387:205-216. https://doi.org/10.1056/NEJMoa2206038 35658024

