# Supplementary material for: Leptin levels are associated with coronary artery calcification in patients with advanced prostate cancer
Source: Oncologist. 2024 Nov 18;30(8):oyae308. doi: 10.1093/oncolo/oyae308 (PMC12395245; doi:10.1093/oncolo/oyae308)
Supplement: oyae308_suppl_Supplementary_Tables_S1-2 [file oyae308_suppl_supplementary_tables_s1-2.docx]

Supplementary Material

Supplementary Table 1: Correlation of measures of adiposity with leptin levels

|  | Pearson correlation coefficients (p-value)  N |
| --- | --- |
|  | Leptin (ng/ml) |
| TAT_index (cm^2^/m^2^) | 0.55 (p<.0001) 152 |
| VAT_index (cm^2^/m^2^) | 0.46 (p<.0001) 152 |
| SAT_index (cm^2^/m^2^) | 0.47 (p<.0001) 152 |
| SMM index (cm^2^/m^2^) | 0.02 (p=0.7926) 152 |
| SMD (HU) | -0.23(p=0.0048) 149 |
| IMA_index (cm^2^/m^2^) | 0.19 (p=0.0200) 152 |

TAT: total adipose tissue volume, VAT: visceral adipose tissue volume, SAT: subcutaneous adipose tissue volume, SMM: skeletal muscle mass, SMD: skeletal muscle density, and IMA: intramuscular adipose tissue

Supplementary Table 2: Association of demographic and clinical characteristics with the number of calcified coronary arteries as a response variable

|  | Univariate linear regression | | Multivariate linear regression^1^ (n=152) | |
| --- | --- | --- | --- | --- |
| Parameter | Estimate (95% CI) | P-value | Estimate (95% CI) | P-value |
| Age at registration | 0.07(0.04-0.10) | **<.0001** | 0.06(0.04-0.09) | <.0001 |
| BMI | 0.02(-0.02-0.07) | 0.3386 |  |  |
| Leptin (ng/ml) | 0.14(0.04-0.24) | **0.0081** | 0.14(0.05-0.24) | 0.0037 |
| SMM index | -0.01(-0.04-0.02) | 0.6353 |  |  |
| SMD_HU | -0.04(-0.07--0.01) | **0.0027** |  |  |
| IMA index | 0.07(-0.01-0.16) | 0.0801 |  |  |
| VAT index | 0.01(0.00-0.01) | **0.0116** |  |  |
| SAT index | 0(-0.01-0.01) | 0.9998 |  |  |
| TAT index | 0(0.00-0.01) | 0.1007 |  |  |
| A1C | -0.16(-0.43-0.12) | 0.2627 |  |  |
| LDL | 0(-0.01-0.01) | 0.8820 |  |  |
| Cholesterol | 0(-0.01-0.01) | 0.8251 |  |  |
| HDL | 0(-0.02-0.03) | 0.9384 |  |  |
| Triglyceride | 0(-0.01-0.01) | 0.6749 |  |  |
| Troponine | -0.01(-0.04-0.02) | 0.5429 |  |  |
| NTproBNP | 0(0.00-0.00) | 0.8550 |  |  |
| Creatinine | 0.35(-0.66-1.37) | 0.4917 |  |  |
| Albumin | -0.86(-1.70--0.02) | **0.0450** |  |  |
| CRP | 0(0.00-0.00) | 0.8984 |  |  |
| PSA | 0(0.00-0.00) | 0.1792 |  |  |
| HTN vs. No | 0.61(0.16-1.05) | **0.0076** |  |  |
| HLD vs. No | 0.59(0.15-1.03) | **0.0084** | 0.52(0.09-0.95) | 0.0171 |
| T2DM vs. No | 0.4(-0.16-0.96) | 0.1595 |  |  |

^1^ Multivariate linear regression model is selected by stepwise selection, initially including age, leptin, SMU_HU, TAT index, HTN, HLD, and albumin.
